# Whole Tumor Antigen Vaccines: Where Are We?

**DOI:** 10.3390/vaccines3020344

**Published:** 2015-04-23

**Authors:** Cheryl Lai-Lai Chiang, George Coukos, Lana E. Kandalaft

**Affiliations:** 1 Ovarian Cancer Research Center, University of Pennsylvania Medical Center, Philadelphia, PA 19104, USA; E-Mail: George.coukos@chuv.ch; 2 Ludwig Cancer Center, University of Lausanne, Lausanne 1011, Switzerland; 3 Center of Experimental Therapeutics, Ludwig Cancer Center, University of Lausanne, Lausanne 1011, Switzerland

**Keywords:** vaccines, dendritic cells, whole tumor, personalized vaccines

## Abstract

With its vast amount of uncharacterized and characterized T cell epitopes available for activating CD4^+^ T helper and CD8^+^ cytotoxic lymphocytes simultaneously, whole tumor antigen represents an attractive alternative source of antigens as compared to tumor-derived peptides and full-length recombinant tumor proteins for dendritic cell (DC)-based immunotherapy. Unlike defined tumor-derived peptides and proteins, whole tumor lysate therapy is applicable to all patients regardless of their HLA type. DCs are essentially the master regulators of immune response, and are the most potent antigen-presenting cell population for priming and activating naïve T cells to target tumors. Because of these unique properties, numerous DC-based immunotherapies have been initiated in the clinics. In this review, we describe the different types of whole tumor antigens that we could use to pulse DCs *ex vivo* and *in vivo*. We also discuss the different routes of delivering whole tumor antigens to DCs *in vivo* and activating them with toll-like receptor agonists.

## 1. Introduction

Whole tumor antigen with its vast amount of characterized and uncharacterized T cell epitopes available for activating CD4^+^ T helper (Th) and CD8^+^ cytotoxic lymphocytes (CTLs) simultaneously, represents an attractive alternative source of antigens to tumor-derived peptides and full-length recombinant tumor proteins for dendritic cell (DC)-based immunotherapy [1]. Although it is relatively easy to synthesize large quantities of clinical-grade tumor-associated peptides for the clinics, most of the identified peptides are human leukocyte antigen (HLA)-A2-restricted which means that mostly HLA-A2 positive patients would benefit from this form of treatment. Moreover, the elicited immune responses in cancer patients are restricted to the peptide used for immunization and might be insufficient for controlling tumor growth. As tumor cells frequently undergo high rates of mutation which could result in the loss of a single or multiple antigens, it would be ideal to choose a source of antigen that can elicit a broad polyclonal tumor-specific response directed against multiple antigenic epitopes. Whole tumor antigen offers this distinct advantage as it allows DCs to process and present numerous tumor antigens to stimulate a strong polyclonal T cell response to prevent tumor escape. The stimulated CD4^+^ Th cells could also provide cognate help to CD8^+^ T cells to generate a more robust anti-tumor immunity and long-term memory. Moreover, whole tumor cell lysate treatment is suitable for all cancer patients regardless of their HLA type. Whole tumor antigen could be administered alone as whole tumor cell lysate, exosomes, whole tumor or tumor stroma RNA, or crude eluted tumor-associated peptides to target DCs *in vivo* or pulsed onto DCs *ex vivo* and subsequently administrated to patients. DC-based immunotherapy offers an exciting opportunity for specific targeting of cancers. DCs are essentially the master regulators of immune responses, and they are the most potent antigen-presenting cell (APC) population for priming and activating naïve T cells to recognize and combat tumors. DCs are heterogeneous and different subsets of DCs could be targeted for tumor immunotherapy based on their unique toll-like receptor (TLR) expressions. Here, we describe the different types of whole tumor antigens available for pulsing DCs *ex vivo* and *in vivo*, and we discuss the routes of administering whole tumor antigen to DCs and activating them with TLR agonists *in vivo*.

## 2. Types of Whole Tumor Antigens

### 2.1. Whole Tumor Cell Lysates

Results from a meta-analysis of about 1,800 patients showed that patients who were immunized with whole tumor vaccines had a significantly higher objective response (8.1%) than patients who were immunized with defined tumor antigens (3.6%) [2]. These results provide a strong rationale for using whole tumor cell lysate for cancer vaccination. Both autologous and allogeneic whole tumor cell lysates can be used for immunizing patients. Allogeneic tumor cell lines that share one or more tumor antigens as autologous tumor cells can be processed easily in large quantities and stored as “off-the-shelf” vaccines. A tumor vaccine that is composed of three irradiated allogeneic melanoma cell lines demonstrated promising results in a phase II clinical trial but was terminated in a phase III study due to low efficacy [3,4]. Another example is the Prostate GVAX vaccine, which consists of allogeneic prostate tumor cell lines LNCaP and PC-3 modified by adenovirus to secrete granulocyte-macrophage colony-stimulating factor (GM-CSF) to enhance the recruitment of DCs to the vaccination site. It has been tested in patients with hormone-refractory prostate cancer (HRPC) in many phase I/II studies. The Prostate GVAX vaccine which demonstrated encouraging results was irradiated before intradermal administration [5,6,7]. In a multicenter trial, 34 of 55 patients with metastatic HRPC treated with Prostate GVAX showed an improved overall median survival of 26 months compared to patients who received taxane chemotherapy alone [8,9,10]. The GVAX approach was also being tested with autologous tumor cells in several phase I clinical trials for stage IV melanoma, advanced ovarian cancer, non-small cell lung cancer, renal cancer and acute myleogenous leukemia. Tumor-infiltrating lymphocytes (TILs) with CTL activity and humoral responses were detected in some of the treated subjects [11,12,13,14], and approximately 20% of the subjects showed a long-term survival of more than five years [15]. Similarly, irradiated autologous whole tumor lysate could be administered in combination with recombinant GM-CSF to cancer patients. Powell and colleagues immunized 22 mesothelioma patients with this treatment method and showed that seven patients developed anti-tumor responses. The treatment was well tolerated with no toxicity, and the median survival of all the patients was 11.5 months. The one- and two-year survival rates were 50% and 27%, respectively [16]. The use of autologous tumor cells is more applicable than allogeneic tumor cell lines as it is a completely personalized approach where the unique array of mutated tumor antigens in each patient could be essential for tumor-rejection responses. Recent data demonstrate that tumor antigens resulting from somatic mutations or epigenetic deregulations are rarely shared among different tumors and actually exhibit an extreme degree of heterogeneity. For example, in ovarian carcinoma, the Cancer Genome Atlas’s (TCGA) data demonstrated an average of 60 private, non-synonymous mutations per tumor [17], thus warranting the personalized whole tumor lysate vaccine approach.

### 2.2. Approaches to Preparing Whole Tumor Cell Lysate Vaccines

Whole tumor cell lysates can be prepared by ultraviolet B (UVB) ray-irradiation or repeat cycles of freezing and thawing, which are the two most commonly used methods in the clinic. Tumor cells that are subjected to UVB-irradiation will undergo apoptosis leading to the exposure of phosphatyidylserine (PS) on the tumor cell surface to facilitate uptake and cross-presentation by DCs [18,19,20]. During cellular apoptosis, calreticulin (CRT) which is a Ca^2+^-binding protein in the endoplasmic reticulum would translocate to the tumor cell surface to act as a crucial determinant for phagocytosis by DCs and macrophages [21]. In addition, high mobility group box 1 [HMGB1] [22,23] and pentraxin-3 [PTX3] [24] are released by late-stage apoptotic cells to stimulate DC maturation and modulate immune responses [25]. Necrosis, for example, as induced by a repeated freeze-thaw process, causes tumor cells to release HMGB1 as well as other cellular components such as heat shock proteins (HSP) 70 and 90, mitochondria, cellular membrane, RNA, DNA and uric acid. DCs have been shown to undergo partial maturation upon interaction with necrotic tumor cells [26]. One possible mechanism is via the recognition of HSP [27] and HMBG-1 [28] by scavenger receptor-A and TLR4 on DCs, and another mechanism is through interaction with uric acid which is the natural end product of the purine metabolic pathway of degraded cellular RNAs and DNAs [29,30]. Hyperthermia, which is the heating of tumor cells, is also a potential method for increasing the immunogenicity (reviewed in [31]). *Ex vivo* heating of tumor cells at 39.5 °C for 6 h could be used to increase the surface expressions of MHC class I-related chain A (MICA) which is a natural killer group 2D (NKG2D) ligand, thus making tumor cells more sensitive to cytolysis by natural killer [NK] cells [32]. Furthermore, the *ex vivo* heating of tumor cells at 42 °C for 30 min enhances their tumor antigen expression leading to better recognition by tumor-specific CD8^+^ T cells [33]. Heating could also upregulate HSPs that have strong immunostimulatory properties. For example, HSP70 released from heated tumor cells could bind directly to TLR4 on DCs to activate their proinflammatory cytokine production and antigen uptake [34].

**Figure 1:**
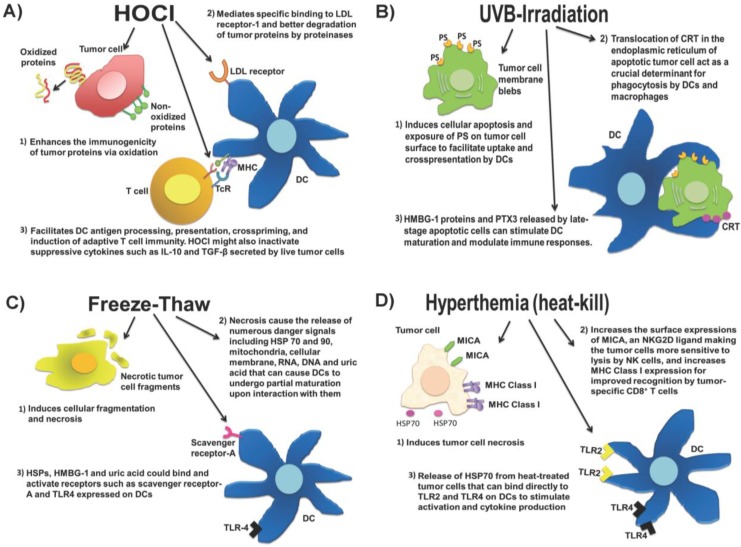
Four whole tumor lysate preparations currently in use in the clinics. (**A**) Hypochlorous acid (HOCl) induces rapid necrotic cell death and enhances the immunogenicity of tumor proteins via oxidation. The increased immunogenicity of HOCl-oxidized tumor proteins are due to a number of mechanisms, including specific binding of oxidized proteins to low-density lipprotein (LDL) receptor to activate dendritic cells (DCs), improved degradation of oxidized proteins by proteinases, improved DC antigen processing and presentation via the major histocompatibility (MHC) molecules, and crosspriming of tumor-specific T cells via T cell receptor (TcR). HOCl might also inactivate IL-10 and transforming growth factor (TGF)-β that can inhibit anti-tumor responses and facilitate T regulatory (Treg) cell proliferation; (**B**) Ultra-violet B (UVB)-irradiation induces apoptotic tumor cell death. This causes the exposure of phosphatidylserine (PS) and translocation of calreticulin (CRT) to facilitate DC uptake and crosspresentation of the apoptotic tumor cells. The release of high mobility box group-1 (HMGB-1) and pentraxin-3 (PTX-3) by late-stage apoptotic tumor cells can stimulate DC maturation and elicit specific anti-tumor T cell responses; (**C**) Repeated freeze-thaw treatment of tumor cells induces necrotic cell death and cause the release of numerous danger signals such as HMGB-1, heat-shock proteins (HSPs), DNA, RNA and uric acid that stimulate partial DC maturation. Danger signals such as HSPs, HMGB-1 and uric acid could bind to scavenger receptor-A and toll-like receptor (TLR) 4 on DCs to elicit immune responses; (**D**) Hyperthermia, which is heat-kill treatment of tumor cells, induces necrotic cell death and increased expression of MICA (major histocompatibility class I chain-related; natural killer group 2D [NKG2D] ligand) and MHC Class I to improved natural killer (NK) cell and antigen-specific CD8^+^ T cell recognition, respectively.

Live tumor cells are shown to produce immunosuppressive cytokines such as IL-10 and TGF-β to hinder anti-tumor T cell responses and promote T regulatory (Treg) cell functions [35]. TGF-β could also inhibit DC differentiation [36,37] and NK cell functions [38]. Therefore, a whole tumor lysate preparation that simultaneously induces an immunogenic cell death and inactivates immunosuppressive cytokine production from the tumor cells would be highly desirable. In a small-scale phase I recurrent ovarian cancer clinical trial at the University of Pennsylvania, we used hypochlorous acid (HOCl) to induce primary necrosis in the tumor cells as well as increase their immunogenicity for DC uptake and processing [1,39,40]. We optimized this approach in a preclinical mouse ID8 ovarian tumor model by comparing the efficacy of DCs loaded with three different whole tumor lysate preparations, *i.e.*, UVB-irradiated tumor lysate, freeze-thawed lysate and HOCl-oxidized whole tumor lysate. We demonstrated that mice treated with bone marrow-derived DCs pulsed with HOCl-oxidized whole tumor cell lysate of ID8 expressing ovalbumin (ID8-ova) had the best tumor control with >60% cure rate [41]. In contrast, mice that were treated with DCs pulsed with freeze-thawed (100%) and UVB-irradiated ID8-ova whole tumor cell lysate (70%), succumbed to tumor growth and ascites formation. The superiority of HOCl-oxidized whole tumor cell lysate preparation could be attributed to the induction of less Treg cells in peripheral blood and absence of serum IL-10 in the vaccinated mice and not in mice treated with other UVB-irradiated or freeze-thawed whole tumor lysate preparations. We translated these findings into a phase I trial by immunizing patients with autologous DCs loaded with autologous whole tumor cell lysate prepared with HOCl-oxidation [41]. Similar to the ID8 mouse ovarian tumor model, we observed that patients who demonstrated strong anti-tumor T cell responses also showed less peripheral Treg cells and reductions of IL-10 in the sera following immunization. In addition, the monocyte-derived DCs loaded with HOCl-oxidized whole tumor cell lysate produced significantly higher IL-12 upon stimulation with LPS and IFN-γ compared to monocyte-derived DCs loaded with UVB-irradiated or freeze-thawed whole tumor cell lysates. As HOCl-oxidation induces necrotic tumor cell death [39], it could also cause the release of numerous danger signals such as DNA, RNA, ATP, uric acid, HMGB1, and HSP from the oxidized tumor cells to increase their immunogenicity and to activate DCs. In addition, numerous HOCl-oxidized biomolecules including proteins, lipids, and glycoproteins could simultaneously engage and activate various scavenger receptors such as LOX-1 [42], CD36, and MARCO, and possibly TLRs present on DCs perhaps leading to higher IL-12 secretion from DCs. Our group and others have shown that ovarian tumors produced IL-10 and TGF-β [43,44], thus it is reasonable to suggest that HOCl-oxidation and not UVB-irradiation or repeat freeze-thaw cycles might have helped to inactivate these suppressive cytokines in the whole tumor cell lysate. Figure 1 demonstrates the current whole tumor lysate preparations used in clinics.

Whole tumor cells can be genetically modified to produce cytokines to enhance the stimulation of anti-tumor response or to inhibit tumor cell production of immunosuppressive cytokines such as TGF-β. Senzer and colleagues recently described a novel FANG vaccine whereby autologous whole tumor cells are being genetically engineered to express GM-CSF via a plasmid and a bifunctional short hairpin RNAi (bi-shRNAi) to target furin convertase to downregulate endogenous TGF-β1 and -β2 [45]. Forty-two patients with advanced cancer received the FANG vaccine intradermally at a dose of 1 × 10^7^ or 2.5 × 10^7^ cells/mL injection once a month for a total of 12 months. The vaccine was well tolerated and only grade 1–2 adverse events such as local induration (*n* = 14) and erythema (*n* = 11) were observed. Tumor-specific responses were detected four months after the last vaccination in 9 out of 18 patients and they positively correlated with the duration of patient’s survival from the time of vaccination (*p* = 0.025). Based on these promising results, a phase II trial with the FANG vaccine is currently being planned. Downregulating TGF-β and increasing GM-CSF secretion would help to enhance the recruitment of DCs and increase the activation of NK cells and tumor-specific T cells. Similarly, autologous whole tumor cells could be transduced with the CD86 (B-7) molecule to increase the priming of anti-tumor T cell responses. Such CD86-expressing irradiated autologous tumor cell vaccines have been evaluated in combination with subcutaneous IL-2 administration in a phase II study involving 66 stage IV renal cell carcinoma patients [46]. These patients received three subcutaneous tumor cell vaccine injections at four-week intervals followed by subcutaneous IL-2 treatment for six weeks starting at week 7. The rationale was to costimulate tumor-reactive T cells with CD86 before IL-2 exposure. It was found that 3% of the patients experienced complete responses, 5% had partial responses, 64% showed stable diseases, and 28% had disease progression after treatment. The median survival of these patients was 21.8 months (95% confidence interval 17.8 to 29.6). Moreover, longer median survival was observed in patients whose biopsies showed lymphocytic infiltration compared to patients whose biopsies lacked such infiltration (28.4 *versus* 17.8 months, *p* = 0.045, two-sided log-rank test, post-hoc comparison).

### 2.3. Exosomes Derived from Tumor Cells

In normal physiological and pathological conditions, almost all cell types including DCs produce exosomes in their multivesicular endosomes, which are subsequently secreted via exocytosis into the extracellular environment. Abundant tumor-derived exosomes are present in the plasma, ascites and pleural effusions of cancer patients, and they resemble miniatures of the host tumor cells by containing a host of tumor-associated antigens such as HER-2/neu, EGFR2, CEA, MART-1, gp100, TRP-1, mesothelin, and members of the HSP family including HSP 70 and HSP 90 [47,48,49,50]. Interestingly, tumor-derived exosomes obtained from stressed tumor cells are shown to be highly immunogenic [51,52], possibly due to the enrichment of danger signals such as uric acid and HMGB1 in the vesicles under stress condition. It could be envisioned that an approach to introduce stress, e.g., HOCl-oxidation, UVB-irradiation or hyperthermia as described earlier, could be used to prepare exosomes from treated tumor cells for loading DCs *in vivo*. On the other hand, increasing evidences suggest that tumor-derived exosomes could suppress T cell by inducing apoptosis *in vitro* through membrane-bound CD95 ligand (FasL) [53] or galectin 9 [54]. Membrane-bound TGF-β on tumor-derived exosomes could also inhibit IL-2 induced T cell proliferation and promote Treg cell function [55]. Furthermore, tumor exosomes could reduce the cytotoxic capacity of NK cells by downregulating NKG2D [56], impairing the differentiation of myeloid precursors into DCs [57], and inducing the generation of myeloid-derived suppressor cells [MDSC] [58]. Tumor-derived exosomes represent an attractive cell-free approach to tumor immunotherapy; however, careful optimization is required to harness their immunostimulatory capability while avoiding the suppressive effects. The use of autologous ascites-derived exosomes (Aex) has been explored in a phase I colorectal cancer clinical trial whereby 40 advanced stage patients were vaccinated subcutaneously once a week for a total of four weeks with Aex alone or in combination with recombinant GM-CSF [59]. The exosomes were found to contain diverse immunomodulatory markers and carcinoembryonic antigen (CEA), which was expressed by all the patients in the study. Aex vaccination was found to be feasible, safe and well tolerated, and patients who received Aex with GM-CSF but not Aex alone showed a beneficial tumor-specific CTL response. In the meantime, more clinical trials are needed to determine if this approach is applicable to other cancer types, and whether the use of exosomes derived directly from tumor cells will also elicit beneficial anti-tumor response.

### 2.4. Tumor Cell-Derived Messenger Ribonucleic Acid

Immunizing cancer subjects with ribonucleic acid (RNA) derived from autologous tumor cells is a promising immunotherapeutic approach. RNAs offer similar advantages as the whole tumor cell lysate; this form of therapy has no HLA-restriction, multiple tumor-associated antigens can be targeted at the same time, and both CD4^+^ and CD8^+^ T cell responses can be elicited via DCs. Most importantly, RNAs can be used when obtaining a large number of tumor cells for creating a whole tumor cell lysate vaccine is prohibitive. Another advantage of RNA is that one can also obtain antigens associated with tumor stroma as increasing evidence now points to the immunomodulatory effects of tumor stroma on anti-tumor responses [60]. RNA are also easily degraded and cleared quickly out of the organism to minimize the chance of causing side effects such as autoimmune disease or generation of autoantibodies. Furthermore, the administration of RNAs is relatively easy as they only need to gain access to the cell cytoplasm as opposed to the cell nucleus by naked DNA. Moreover, large amounts of RNA can be prepared *ex vivo* by generating complete tumor cDNA libraries with polymerase chain reaction (PCR) technology [61,62]. One major disadvantage of RNAs is that they are intrinsically unstable, and careful handling of tumor samples and extracted RNAs is required right from the initial biopsy excision. Due to the technical challenges relating to the handling of RNA to avoid degradation, no clinical trial has been published to our knowledge that described the direct administration of tumor-derived RNAs in patients to target DCs *in vivo*.

Alternatively, electroporation can be used to introduce mRNA rapidly into DCs *ex vivo* to protect against rapid degradation. When modified *ex vivo*, DCs can be made to express, process and present specific tumor antigens to a high level to help elicit a higher avidity tumor-specific T cell response. A number of clinical trials have reported vaccination of cancer patients with DCs that are transfected with tumor RNA (reviewed in [63]). In a phase I trial, autologous DCs were transfected with total renal tumor cell RNA by electroporation to treat 10 patients with metastatic renal cell carcinoma [64]. In this trial, the use of mRNA represents the entire spectrum of tumor-associated antigens that could help in generating a polyclonal tumor response. In 6 of the 7 evaluable patients, the modified DC vaccine elicited a broad-based anti-tumor response directed against many tumor antigens including hTERT, G250 and oncofetal antigen, but not against self-proteins that are expressed by normal renal tissues. It was also noted that 7 out of 10 patients showed a prolonged mean overall follow-up of 19.8 months. In another phase I/II trial, 22 patients with advanced melanoma were vaccinated with monocyte-derived autologous DCs that were transfected with autologous whole tumor mRNA [65]. Antigen-specific T cell proliferation and IFN-γ responses were detected in 9 out of the 19 evaluable patients, while 10 out of 22 patients experienced delayed-type hypersensitivity (DTH) reaction. In a glioblastoma study, patients were treated with autologous monocyte-derived DCs electroporated with the whole mRNA of glioblastoma cancer stem cells [66]. No adverse side effects were observed in the patients following treatment. T cells specific to hTERT- or survivin-derived peptides were detected in all the 7 treated patients, while the progression-free survival was 2.9 times longer in vaccinated patients (median 694 *vs.* 236 days, *p* = 0.0018, log-rank test) than in control patients.

### 2.5. Personalized Mutanome-Based Vaccines

The cancer mutanome, which refers to an individual patient’s tumor-specific mutations and alterations that result in the generation of neo-tumor antigens, can potentially be exploited to treat cancer patients in a highly personalized manner. It has been demonstrated that 95% of the mutations in a patient’s tumor appeared to be unique and only a small number of mutations are shared between patients [67]. It has been suggested that such mutanome-encoded antigens may evoke a more vigorous T cell response than shared tumor antigens due to a lack of thymic tolerance against them. Moreover, mutanome-encoded antigens are restricted to the tumors that express the mutated antigens [68]. The rapid advancement of next-generation sequencing technologies and bioinformatics allow for rapid and affordable interrogation of patients’ genome, exome, epigenome and transcriptome at the single nucleotide level. Indeed, genome-wide mutation identification initiatives have been launched which include the Cancer Genome Association and the International Cancer Genome Consortium to map every patient’s individual tumor mutanome “signature.” This should help in identifying important tumor cell targets.

One potential method of immunizing cancer patients with mutanome-based vaccines is through the administration of coding mRNAs that have been synthesized to encode for multiple types of mutated tumor transcripts [69]. This approach has several advantages as we could customize the mRNAs to increase their therapeutic value by selecting the sequences that encode for highly immunogeneic mutated tumor antigens. Other potential approaches include the use of directly eluted tumor-associated peptides from the tumor cell surface and circulating tumor-derived HLA-peptide complexes [70]. Using the murine B16.F10 melanoma cell line for cancer mutanome analysis, Castle and colleagues sequenced the protein-coding genome (*i.e.*, the exome) of B16.F10 cells and of C57BL/6 wild-type mouse cells [71,72]. It was found that of the 50 confirmed expressed non-synonymous mutations, one-third of these mutated epitopes were as strongly immunogenic as the positive control TRP-2 which is the strongest known B16.F10 murine melanoma antigen. Moreover, the induced T cell response was specific to the mutated epitopes and not to the respective wild types [73]. Similarly, Robbins and colleagues evaluated three melanoma patients who responded to the treatment with *ex vivo* expanded autologous tumor-infiltrating lymphocytes (TILs), and discovered a total of seven unique mutanome-derived tumor peptides that were presented by autologous tumor cells and recognized by the autologous TILs [74]. In a clinical study, nearly 50% of the patients with vulvar intraepithelial neoplasia experienced complete remissions after treatment with neo-epitopes of human papilloma virus (HPV) E6 and E7 peptides [75]. Moreover, several clinical studies have tested mutated epitopes or individually expressed mutations as vaccine targets [76,77,78] and demonstrated that mutated epitopes could elicit immune responses in the immunized patients [79] and could act as tumor-rejection antigens in mouse tumor models [80]. In the case of advanced melanoma, it has been shown that the induction of a poly-epitopic tumor-specific immune responses by autologous patient-specific vaccines correlated with complete remissions [81]. Thus, interrogating the cancer mutanome would potentially provide numerous targets for poly-epitope vaccine development in each individual patient. Phase I clinical trials have been initiated to test mutanome-derived tumor peptides in glioblastoma (ClinicalTrial.gov: NCT02287428) and melanoma (NCT01970358, NCT02035956). A summary of vaccine studies utilizing whole tumor antigen in preclinical animal models and in the context of clinical trials are summarized in Table 1 and Table 2 respectively.

### 2.6. Tumor Stroma-Associated Antigens

Besides targeting tumor cells directly, increasing attention has been given to target the tumor stroma where heterogeneous populations such as fibroblasts, endothelial cells, T reg cells, tumor-derived macrophages (TAMs), MDSCs, as well as extracellular matrix could be found. Together, they help to create a highly immunosuppressive tumor microenvironment for tumorigenesis, progression and metastasis. Unlike tumor cells that exhibit high rates of somatic mutations, tumor stroma cells are genetically more stable thus making them attractive targets for tumor-specific T cells to induce long-term tumor control. Moreover, tumor cells could employ numerous evasion mechanisms, e.g., defects in their antigen-presentation pathways due to loss of MHC class I heavy chains, β2-microglobulin and/or tapasin expression, to prevent tumor-specific CTL recognition. Tumor stroma cells, on the other hand, are less subjected to such immune evasion mechanisms.

Tumor stroma cells are shown to be different from their normal counterparts owing to their upregulation of certain tumor stroma-associated antigens (TSAAs) under the influence of the tumor microenvironment. The cancer-associated fibroblasts (CAFs), being the most abundant cell type in the stroma, could serve as useful targets as they are involved in multifaceted functions such as remodeling the tumor extracellular matrix, suppressing anti-tumor immune responses, and secreting growth factors and cytokines that promote tumor growth, invasion, differentiation, angiogenesis, and chronic inflammation (reviewed in [82]). CAFs overexpressed fibroblast activation protein α (FAPα; seprase) which is a surface glycoprotein that is only induced during carcinogenesis in numerous solid tumors [83,84,85]. Its overexpression is associated with increased tumor growth, invasion, angiogenesis, metastasis [86,87], and reduced survival in patients with FAPα^+^ gastric carcinoma [83]. In addition, the matrix metalloproteinases (MMPs) and their inhibitors are TSAAs that are expressed by CAFs, endothelial cells, TAMs and tumor cells. They are overexpressed in neoplasia and are critical modulators of the extracellular matrix composition.

**Table 1:** Evaluation of different whole tumor antigens in selected preclinical mouse tumor models.

Source of Whole Tumor Antigen	Method of Whole Tumor Antigen Preparation	Treatment Regimen	Outcome of Study	Reference
Whole mouse glioblastoma cell lysate	Freeze-thawed whole tumor lysate administered alone or in combination with type B CpG (ODN 1826)	Glioblastoma-bearing mice were immunized subcutaneously with the tumor lysate-CpG vaccine	Significantly more T cells and activated DCs were observed in lymph nodes draining the vaccination site of mice that were treated with lysate-CpG when compared to mice treated with CpG or tumor lysate alone (*p* < 0.05) 55% of the mice vaccinated with CpG-lysate vaccine tumor-free and showed 2 times greater median survival than mice that were treated with CpG only, tumor lysate only or no treatment (*p* < 0.05)	[88]
Whole fibrosarcomas or mammary carcinoma cell lysate	Freeze-thawed whole tumor cell lysate was pulsed onto mouse bone marrow-derived DCs	Fibrosarcomas or mammary carcinoma-bearing mice were vaccinated subcutaneously with the whole tumor lysate-DC vaccine	Both CD8^+^ and CD4^+^ tumor-specific T cells were induced following vaccination that contributed to tumor suppression Significant reductions in the number of lung metastases were observed in mice treated with the whole tumor lysate-DC vaccine when compared to mice treated with tumor lysate alone, unpulsed DC alone or untreated	[89]
Whole ovarian carcinoma cell lysate	Whole tumor cells were oxidized with HOCl and then frozen and thawed, and pulsed onto mouse bone marrow-derived DCs (*i.e.*, mouse OCDC vaccine)	Ovarian carcinoma-bearing mice were treated intradermally with OCDC vaccine	Mice treated with OCDC had the best tumor control with >60% cure rate, while 100% and 70% of the mice treated with DCs pulsed with freeze-thawed or UVB-irradiated lysate succumbed to tumor growth and ascites Less peripheral Treg cells and absence of serum IL-10 were observed in mice treated with OCDC vaccine when compared to mice treated with DC vaccine pulsed with UVB-irradiated or freeze-thawed tumor lysate	[41]
Whole ovarian tumor cells expressing HPV E6 and E7	UVB-irradiated whole cells pulsed onto mouse bone marrow-derived DCs	Ovarian carcinoma-bearing mice were treated intraperitoneally and subcutaneously with the UVB-irradiated tumor cell-DC vaccine	E6- and E7-specific IFN-γ secreting T cells were elicited that infiltrated the tumor and helped suppressed tumor growth	[90]
Total RNA of whole ovarian tumor cells expressing HPV E6 and E7	Total tumor RNA was electroporated into mouse bone marrow-derived DCs	Ovarian cancer-bearing mice were vaccinated with DCs electroporated with total tumor mRNA via the intraperitoneal and subcutaneous routes	It was found that DCs loaded with total tumor RNA elicited a significantly higher IFN-γ secreting E6- and E7-specific T cells than DCs loaded with UVB-irradiated whole tumor cells in the mice DCs electroporated with total tumor RNA also induced a larger tumor infiltration by T cells and produced a significantly stronger delay in tumor growth when compared to DCs pulsed with UV-irradiated tumor cells	[90]
Mutanome peptides of mouse melanoma cells	Mutated epitopes of tyrosinase-related protein 2 (TRP-2) were identified via sequencing the protein-coding genome of the B16.F10 mouse melanoma cells	Melanome-bearing mice were immunized subcutaneously with the long mutanome peptides and Poly(I:C) as adjuvant	A total of 50 validated mutations were found to be immunogenic following immunization of the mutated epitopes in the mice Melanoma-bearing mice immunized with the mutated peptides successfully controlled tumor growth	[73]

**Table 2:** Evaluation of different whole tumor antigens in selected published clinical trials.

Source of Whole Tumor Antigen	Method of Whole Tumor Antigen Preparation	Treatment Regimen	Outcome of Study	Reference
Autologous whole ovarian tumor cells	Whole tumor cells were modified with dinitrophenyl (DNP) and UVB-irradiated	Phase I trial in stage III ovarian cancer where patients were immunized intradermally with the vaccine	No acute toxicities. Anti-tumor responses were demonstrated in some patients but no clinically meaningful responses were observed.	[91]
Autologous whole melanoma cells	Whole tumor cells were UVB-irradiated	Phase III/IV metastatic melanoma trial where patients were vaccinated intradermally with UVB-irradiated autologous whole cells and Bacillus Calmette-Guérin (BCG) as adjuvant	5-year overall patient survival rate was ~45% which was superior to historical controls (at 35% and 20% for Stage III and IV, respectively).	[92]
Allogeneic melanoma cell lysate derived from 3 different melanoma lines (TRIMEL)	TRIMEL was subjected to freeze-thawed cycles and pulsed on autologous monocyte-derived DCs (*i.e.*, TRMEL-DC vaccine)	Phase I trial where stage IV and III melanoma patients were vaccinated intradermally with TRIMEL-DC vaccine and aluminum hydroxide/keyhole limpet haemocyanin (KLH) as an adjuvant	>60% of the stage IV patients experienced positive DTH reaction after treatment Median survival of these patients was 33 months compared to 11 months for stage IV patients without DTH response. All stage III patients were DTH-positive after vaccination and remained tumor-free for a median follow-up period of 48 months (range of 33 to 64 months)	[93]
Allogeneic whole prostate tumor cells	Prostate tumor cell lines LNCaP and PC-3 were genetically modified to secrete GM-CSF (*i.e.*, GVAX vaccine) and UVB-irradiated	Phase I/II studies in metastatic hormone-refractory prostate cancer (HRPC) whereby patients were immunized intradermally with GVAX vaccine	In the largest of these multicenter trials, 34 of 55 HRPC patients demonstrated an improved overall median survival of 26 months that compared favorably with the median survival observed in similar HRPC patients in phase II taxane chemotherapy trials	[5,6,7]
Autologous whole mesothelioma tumor cells	Autologous tumor cell lysate was administered with recombinant GM-CSF	Phase I trial of mesothelioma patients who were vaccinated subcutaneously with whole tumor lysate vaccine and recombinant GM-CSF	Antitumor immune response and was detected in 7 out of 22 immunized patients. The median survival of all the patients was 11.5 months. 1- and 2-year survival rates were 50% and 27%, respectively.	[16]
Autologous whole ovarian tumor cell lysate	Whole tumor cells were oxidized with HOCl and then frozen and thawed, and pulsed on autologous monocyte-derived DCs (*i.e.*, OCDC vaccine)	Phase I trial of recurrent ovarian cancer whereby patients were vaccinated intranodally with OCDC vaccine	Induction of tumor-specific IFN-γ T cell responses, and reductions of peripheral Tregs and serum IL-10 observed in 4 out of the 5 immunized patients 2 out 5 immunized patients achieved prolonged progression-free survival of over 2 years	[41]
Autologous Ascites-derived exosomes (Aex) from colorectal tumor cells	Aex administered alone or in combination with recombinant GM-CSF	Phase I colorectal cancer whereby advanced stage patients are vaccinated subcutaneously with Aex ± recombinant GM-CSF.	Aex vaccination was found to be safe and well tolerated Beneficial tumor-specific CTL response was observed in patients who received Aex with GM-CSF but not Aex alone	[59]
Total mRNA derived from renal tumor cells	Total tumor mRNA was used to transfect autologous monocyte-derived DCs	Phase I trial of metastatic renal cell carcinoma where patients were vaccinated intravenously with the tumor mRNA-expressing DCs	Polyclonal anti-tumor response directed against many tumor antigens including hTERT, G250, and oncofetal antigen were detected in 6 of the 7 evaluable patients following immunization with the total tumor mRNA-expressing DCs 7 out of 10 immunized patients showed a prolonged mean overall follow-up of 19.8 months	[64]
Total mRNA derived from melanoma cells	Total tumor mRNA was used to transfect autologous monocyte-derived DCs	Phase I/II trial of advanced melanoma where patients were vaccinated intradermally or intranodally with the tumor mRNA-expressing DCs. No serious adverse effects were observed.	A vaccine-specific IFN-γ response was demonstrated in 9 out of 19 evaluable patients 7 out of 10 patients vaccinated intradermally showed DTH reaction, while 3 out of 12 patients vaccinated intranodally had DTH reaction	[65]
Total mRNA derived from glioblastoma cancer stem cells	Total mRNA from cancer stem cells was electroporated into autologous monocyte-derived DCs	Phase I trial where patients were treated intradermally with autologous monocyte-derived DCs that were electroporated with the total mRNA of glioblastoma cancer stem cells	T cells specific to hTERT or survivin were detected to all the 7 treated patients Progression-free survival was 2.9 times longer in vaccinated patients (median 694 *vs.* 236 days, *p* = 0.0018, log-rank test) than in control patients	[66]
Mutanome peptides derived from E6 and E7 of HPV	Synthetic long peptides administered in incomplete Freund’s adjuvant	Phase I study where patients with vulvar intraepithelial neoplasia were treated subcutaneously with the E6 and E7 mutanome peptides	85% of the immunized patients developed T cells that were specific for E6, E7, or both ~50% of the patients experienced complete remissions after treatment with E6 and E7 neo-epitopes	[75]
Mutanome peptides derived from von Hippel-Lindau (VHL) gene mutations in renal cell carcinoma	Synthetic neo-peptides derived from Hippel-Lindau (VHL) gene mutations in RCC	Pilot clinical trial whereby patients with advanced RCC and mutated VHL genes were vaccinated subcutaneously with the relevant VHL peptide mixed with Montanide	The vaccine was well tolerated with no grade III or IV toxicities observed Four out of five evaluable patients showed specific immune responses against the corresponding mutant VHL peptides Median overall survival and median progression-free survival of the patients were 30.5 and 6.5 months, respectively	[79]

Endothelial cells of the tumor stroma have been shown to overexpress a set of tumor endothelial markers (TEMs), such as TEM1 and TEM8, during tumor angiogenesis [94]. Although TEM1 mRNA is ubiquitously expressed on normal human endothelial cells, the TEM1 protein is restricted to the corpus luteum and tissues of healing wounds and tumors. TEM8 protein, on the other hand, is undetectable during wound healing or in the corpus luteum [94,95] but is highly expressed in the endothelial cells of breast, esophagus, lung, and bladder cancer stroma [96]. Prostate-specific membrane antigen (PSMA) is another endothelial cell surface molecule that is upregulated on prostate tumor cells as well as on tumor endothelial cells of the stroma of breast, renal cell, bladder, non-small cell lung, prostate and rectal carcinoma, glioblastoma multiforme and melanoma [97]. We envisaged that whole lysate or whole RNA of tumor stroma could be prepared in a similar manner as with tumor cells, and be administered directly to patients or pulsed onto DCs to elicit therapeutic responses against the characterized as well as uncharacterized TSAAs. Tumor stroma could also be targeted by adoptively transferred effector T cells that recognize tumor antigen-loaded stromal cells. This approach has been tested in a preclinical mouse tumor model and resulted in the killing of stromal CD11b^+^/Gr1^+^ myeloid cells as well as inhibiting long-term tumor growth. Thus, targeting both the tumor cells and tumor stroma could be an effective approach to eradicate established tumors. As certain TSAAs are expressed by a broad spectrum of solid tumors, we could develop tumor stroma-based therapies that are applicable to many cancer types.

## 3. Factors Influencing the Immune Responses to Whole Tumor Antigens

### 3.1. Immunodominant Antigens versus Mutated Neo-Antigens for Vaccination

The identification of tumor-associated antigens (TAAs) with early technology such as SEREX (serological analysis of autologous tumor antigens in serum of cancer patients by recombinant cDNA expression cloning), has allowed us to classify TAAs into five broad categories: (1) mutated antigens only expressed by the tumors; (2) overexpressed normal self-antigens; (3) oncofetal antigens as those expressed in fetal tissues; (4) differentiation or lineage antigens; and (5) cancer-testis antigens that are also expressed by spermatocytes/spermatogonia. Many of the current whole tumor antigen vaccines, whether with allogeneic or autologous tumor as the starting material, are developed based on the knowledge of the human immune system’s ability to recognize immunodominant antigens (*i.e.*, antigens that are most easily recognized by the immune system to elicit antigen-specific T cell and antibody responses. However, only a small number of patients to date have achieved tumor regressions with whole tumor vaccines containing immunodominant antigens. As many of the immunodominant tumor antigens are self-proteins, they are subjected to both central and peripheral tolerances that dampen the elicited immune responses to avoid autoimmunity. Ironically, it is through the use of highly immunogenic cancer vaccine and robust immunization regimen (such as the inclusion of T cell checkpoint blockade and/or anti-angiogenesis) that we can break self-tolerance to achieve tumor control and suppression. As discussed earlier, the TCGA data revealed that cancers from individual patients showed a high degree of unique mutations that are not shared amongst patients who have the same type of cancer. Therefore, an attractive alternative is to prepare personalized autologous whole tumor cancer vaccines that contain the unique mutated neo-tumor antigens of each individual patient (e.g., mutanome-based vaccines). As mutated neo-antigens are presumably not subjected to central tolerance, the elicited immune responses might be more potent than that generated against immunodominant antigens. In addition, these mutated antigens are uniquely expressed by the tumors and therefore should not induce toxicity to the normal tissues. Although there is a possibility that vaccination with a multivalent vaccine (whether containing immunodominant or mutated neo-tumor antigens) could lead to unexpected immunodomination of a single or few antigens, there is still a strong rationale to immunize patients with a multivalent whole tumor cancer vaccine as opposed to single antigen vaccine to target multiple tumor antigens in parallel to prevent tumor escape. One study demonstrated that the numbers of APC such as DCs available for presenting the tumor antigens and IL-12 could help modulate the immunodominance of certain tumor antigens [98]. These factors could be taken into account when designing a DC-based immunotherapy with whole tumor antigen vaccine.

### 3.2. Immune Status of Cancer Patients

The immune status of cancer patients may influence the magnitude of the elicited immune responses to whole tumor antigen vaccine. Nevertheless, most of the subjects who are enrolled in cancer clinical trials are elderly patients who have significantly compromised immune systems specifically when immunosenescence sets in (reviewed in [99]). This is characterized by a lower number of peripheral naïve T cells and reduced diversity in the naïve T cell receptor (TCR) repertoire when compared to younger individuals [100]. Although there is an increase in the number of memory T cells in the elderly population, the diversity and functional integrity of both CD4^+^ and CD8^+^ T cells are reduced resulting in the inability to respond adequately or to retain memory for cancer antigens expressed by relapsing tumors. Certain experimental interventions aiming to boost T cell immunity have been investigated in animal models. An example is implanting genetically engineered stromal cells in the thymus to locally secrete IL-7, a T cell survival factor [101]. An alternative way is through nutritional interventions. A study demonstrated that the use of vitamin E supplement was able to reconstitute immunological synapse formation, especially in the naïve CD4^+^ T cells of old mice [102]. Another study showed that the use of conjugated linoleic acid helped to modulate lipid intake leading to decreased proinflammatory cytokine secretion thus increasing the success rate of hepatitis B vaccination in elderly people [103]. Importantly, the lipid environment could strongly influence T cell function, as well as alter their cell membrane fluidity and immunological synapse formation [104,105]. Cancer patients previously treated with chemotherapy and/or radiotherapy could also experience a transient compromised immune system due to the toxic effects of the treatments to proliferating bone marrow progenitor cells. However, accumulating evidence shows that chemotherapy and radiotherapy could also synergize with tumor immunotherapy resulting in better efficacies in the patients. For example, 17 patients with advanced cancer were immunized with a DNA plasmid vaccine encoding for the tumor antigen CYP1B1 plasmid encapsulated in biodegradable poly-dl-lactide-coglycolide microparticles [106]. Five patients who developed CYP1B1-specific responses and then received chemotherapy showed a greater and more durable response to salvage chemotherapy than similarly treated patients who did not develop CYP1B1-specific immunity. These results suggested that patients who were immunized with cancer vaccine previously could benefit more from subsequent chemotherapy than those patients who were not immunized. Likewise, radiotherapy and tumor immunotherapy could synergize to result in increased efficacies in patients [107,108].

## 4. *In Vivo* Activation of Dendritic Cells (DCs)

The most widely used DC subset for DC-based immunotherapy in the clinic is the myeloid monocyte-derived DC, which is differentiated *ex vivo* with recombinant GM-CSF and interleukin (IL)-4. These DCs are highly efficient in the phagocytosis of antigens and production of high IL-12 upon activation. Although they have been used successfully for treating cancers, there are disadvantages and advantages associated with using them. The major advantage is that we can easily manipulate them before administering them to patients. We can load the DCs with different tumor antigens and activate them with a vast array of adjuvants, including TLRs, Montanide and KLH, to express desired phenotypic markers and cytokines that will enhance their anti-tumor stimulating capacity [109]. On the other hand, one major disadvantage is that the *ex vivo* production of these DCs is labor intensive and costly. An attractive alternative is to specifically target DCs *in vivo* by loading them with appropriate tumor antigens and activating them to produce proinflammatory cytokines to elicit potent anti-tumor T cell responses. One major advantage of *in vivo* targeting is that we can use adjuvants to selectively activate one or more DC subsets via their surface receptors to increase the potency of the anti-tumor responses. One major disadvantage is that we have less control over the quality and magnitude of the induced anti-tumor responses. To successfully target DCs *in vivo*, we would need to determine the optimal type of antigen for loading the DCs, the adjuvant for activating them, and finally the best route of administration.

Different DC subsets are present in the human immune system, and they follow two major pathways of development, *i.e.*, lymphoid-derived pDCs and myeloid-derived conventional DCs that develop from CD34^+^ hematopoietic progenitor cells [110,111]. The myeloid-derived DCs can be further classified into different subsets according to their phenotype, receptor expression, chemokine and cytokine production, tissue location, and the type of immune response they induce. Such myeloid-derived DCs include the Langerhans cells (LCs), dermal DCs (also known as interstitial DCs), and DCs present in the blood and lymph nodes. Thus, different DC subsets can be targeted based on their tissue locations and the route of tumor antigen administration (e.g., intradermal, intranodal or intratumoral). In addition, we can target and activate a specific DC subset via their distinct toll-like receptor (TLR) expressions. Upon TLR stimulation, DCs would produce proinflammatory cytokines and chemokines for priming antigen-specific T cells. For example, pDCs uniquely expressed TLR7 and 9 and can be activated to become potent secretors of IFN-α and -β with Imiquimod and cytosine-phosphateguanine oligodeoxynucleotides (CpG), respectively. LCs can be specifically activated via TLR3 with polyribosinic:polyribocytidylic acid (Poly I:C), which is a TLR3 agonist. Dermal DCs express TLR4 and can be activated by monophosphoryl lipid A (MPLA) which is a chemically modified, less toxic version of the lipopolysaccharides (LPS). Lastly, blood and lymph node DCs expressing TLR8 can be targeted with VTX-2337 and VTX-297 that are novel small molecules TLR8 agonists developed by VentiRx Pharmaceuticals (Seattle, WA, USA).

## 5. *In Vivo* Tumor Antigen Administration: Intradermal, Intranodal and Intratumoral

Subcutaneous and intramuscular have been the traditional routes for vaccinations against many different viral and bacterial diseases. Recent clinical results showed that the intradermal route is more effective than the conventional subcutaneous and intramuscular routes in inducing protective immunity against many diseases, including hepatitis B virus (HBV) [112,113], rabies [114], tuberculosis [115], measles [116], polio [117] and influenza [118]. Moreover, subjects who are unresponsive to the intramuscular route of vaccination for HBV can achieve seroconversion at a lower vaccine dose via intradermal vaccination [119]. A similar observation has been made with the rabies vaccine whereby subjects receiving a lower dose intradermally achieved a long-lasting antibody response that was comparable to subjects receiving a full dose intramuscularly [120]. As the dermis contains a much larger DC population compared to the subcutaneous fat and muscles, it is reasonable to postulate that a larger number of dermal DCs would take up the antigen and migrate to the draining lymph nodes to prime more potent antigen-specific responses. The extensive network of lymphatic vessels found in the dermis could also facilitate the migration and interaction between DCs and T cells for stronger T cell priming. Furthermore, the increase in the local pressure of the interstitial tissue from the fluid introduced by intradermal vaccination could help to increase the lymphatic flow of DC and antigen to the draining lymph nodes for presentation to T cells. Finally, intradermal vaccination might be advantageous as it favors the drainage of antigen via the lymphatic system in the dermis minimizing the risk of systemic shock, which is more likely to occur with intramuscular injections when antigens enter the general blood circulation.

Intranodal administration of tumor antigens into the regional draining lymph nodes is perhaps the most straightforward method of ensuring maximum antigen exposure to DCs. It also offers the advantage whereby DCs do not need to migrate, as opposed with other routes discussed earlier, for they are already in close proximity with T cells in the lymph nodes. LCs and dermal DCs that migrated in the draining lymph nodes of the skin can also be pulsed this way. In a mouse study, Kreiter and colleagues investigated the different routes of administering antigen-coding mRNA. The authors demonstrated that resident DCs in the lymph nodes selectively took up the mRNA following intranodal injection, and induced potent antigen-specific CD4^+^ and CD8^+^ T cell responses in tumor-bearing mice [71,72]. They also showed that potent CTLs and memory T cells were generated in mice via repeated RNA intranodal injections, but not via subcutaneous, intradermal or near nodal administrations. The therapeutic efficacy of this mRNA could be further enhanced by pre-treating mice with a fusion protein consisting of the extracellular domain of human fms-like tyrosine kinase 3 (FLT3) ligand and the heavy chain constant regions 2 and 3 (CH2–CH3 domain) of human immunoglobulin (Ig)-G4. The administration of the FLT3 ligand/IgG4 fusion protein would help in recruiting immature pDCs and enhancing the uptake of the antigen-coding mRNA. The approach is currently being tested in a phase I dose escalation trial in advanced melanoma with the aim of evaluating the safety and tolerability of intranodal administration of tumor antigen-coding mRNA (NCT01684241). We envisage that FLT3 ligand could also be used with whole tumor mRNA to increase their uptake by DCs.

Intratumoral administration of tumor antigens to tumor-infiltrating DCs (TIDCs) and activating them might be an alternative option. TIDCs have been documented in a number of cancers including ovarian, breast, colorectal, lung, renal cell carcinomas and melanoma [121]; however, their functions have not been well characterized. There are some conflicting reports suggesting that immature TIDCs could help in promoting tumor growth, while mature TIDCs helped in eliciting antitumor responses [121]. Nevertheless, a phase I trial of bladder cancer demonstrated that intravesical infusion of *Mycobacterium bovis* bacillus Calmette-Guérin (BCG) onto the bladder wall promoted longer survival and delayed progression of the treated subjects. These effects might be mediated by TIDCs that were activated by infused BCG via their TLR2 [122]. Another phase I trial of recurrent glioblastoma demonstrated that subjects who received intracranial administering of CpG showed induction of antitumor responses possibly via activated TIDCs [123]. More studies are required to characterize the phenotypes and functions of TIDCs, and to determine if they could be activated *in vivo* with different immunomodulatory agonists including TLR agonists. The ability of TIDCs to stimulate a therapeutic antitumor response could be highly dependent upon their state of maturation.

Recently, biodegradable particles have been developed as a mean to effectively deliver antigen and activate DCs *in vivo*. There are different biodegradable particles available ranging from liposomes to acid-degradable hydrogels and synthetic polymers. For example, Poly(lactic-co-glycolic acid) (PLGA)-encapsulated breast cancer tumor lysate vaccine has demonstrated significant reduction in tumor burden when administered subcutaneously to mice [124]. In a prostate cancer mouse model, particles co-encapsulating tumor lysate and CpG could induce potent long-lasting CTL responses upon subcutaneous administration [125]. Another example is the use of mesoporous silica rods (MSRs) as a mean to activate DCs and other immune cells to treat cancers and other diseases. Such MSRs contain small holes known as nanopores that can be loaded with cytokines, chemotherapeutic drugs and/or tumor antigens to attract and activate specific immune cells [126]. The MSRs can be administered easily via injection underneath the skin and spontaneously assemble into a three-dimensional (3D) scaffold at the vaccination site. By controlling the types of cytokines released from the MSRs, we could potentially manipulate the type of DCs being recruited to the 3D scaffold to initiate an immune response. One advantage of MSRs is that they are biodegradable and dissolve naturally within a few months. MSRs can also be manufactured easily and rapidly. MSRs have been tested in mice and are found to be effective in recruiting millions of DCs to the 3D scaffold before migrating to the lymph nodes and triggering a powerful immune response [126]. As demonstrated above, biodegradable particles hold great promise for an *in vivo* vaccination approach and should be investigated further.

## 6. Conclusions

Whole tumor antigens, as opposed to defined tumor-derived peptides and proteins, could represent the ideal source for pulsing DCs as they contain a vast array of immunogenic epitopes to activate both CD4^+^ and CD8^+^ tumor-specific T cells to prevent tumor escape. The use of adjuvants, particularly TLR agonists, is a very promising approach to augment DC-whole tumor antigen vaccination. Distinct subsets of DCs are present in humans, and they exhibit different phenotypic markers and TLR expressions allowing for specific *in vivo* targeting with different TLR agonists. DCs located at different tissue compartments are capable of eliciting different immune responses, thus the selection of DC subset for targeting will depend upon the type of immune response desired.
